# Dietary *Clostridium butyricum* and 25-Hydroxyvitamin D_3_ modulate bone metabolism of broilers through the gut—brain axis

**DOI:** 10.1016/j.psj.2024.103966

**Published:** 2024-06-10

**Authors:** Guangtian Cao, Yang Yu, Huixian Wang, Huijuan Yang, Fei Tao, Shenglan Yang, Jinsong Liu, Zhanming Li, Caimei Yang

**Affiliations:** ⁎ College of Standardisation, China Jiliang University, Hangzhou 310018, PR China; † Key Laboratory of Applied Technology on Green-Eco-Healthy Animal Husbandry of Zhejiang Province, Zhejiang Provincial Engineering Laboratory for Animal Health and Internet Technology, College of Animal Science and Technology, Zhejiang A & F University, Hangzhou 311300, PR China; ‡ Zhejiang Vegamax Biotechnology Co., Ltd, Anji 313300, PR China; § School of Grain Science and Technology, Jiangsu University of Science and Technology, Zhenjiang 212004, PR China

**Keywords:** Clostridium butyricum, vitamin D, bone development, gut–brain axis, broiler

## Abstract

Leg disorders have become increasingly common in broilers, leading to lower meat quality and major economic losses. This study evaluated the effects of dietary supplementation with *Clostridium butyricum* (***C. butyricum***) and 25-hydroxyvitamin D_3_ (**25-OH-D_3_**) on bone development by comparing growth performance, tibial parameters, Ca and P contents of tibial ash, bone development-related indicators’ level, and cecal short-chain fatty acids in Cobb broilers. All birds were divided into four treatment groups, which birds fed either a basal diet (**Con**), basal diet + 75 mg chlortetracycline/kg (**Anti**), basal diet + *C. butyricum* at 10^9^ CFU/kg (**Cb**), basal diet + *C. butyricum* at 10^9^ CFU/kg and 25-OH-D_3_ at 25 μg/kg (**CbD**), or basal diet + 25-OH-D_3_ at 25 μg/kg (**CD**). Our results suggest that the dietary supplementation in Cb, CbD, and CD significantly increased the body weight (**BW**) and average daily gain (**ADG**), and reduced the feed-to-weight ratio (**F/G**) at different stages of growth (*P* < 0.05). Dietary supplementation in Cb, CbD, and CD prolonged (*P* < 0.05) the behavioral responses latency-to-lie (**LTL**) time, reduced (*P* < 0.05) the levels of osteocalcin (**BGP**) and peptide tyrosine (**PYY**), and increased (*P* < 0.05) serotonin (**5-HT**) and dopamine (**DA**). Treatment with Cb increased (*P* < 0.05) the levels of acetic acid, isobutyric acid, butyric acid, and isovaleric acid compared with those in Con group. The cecal metagenome showed that *Alistipes* spp. were significantly more abundant in Cb, CbD, and CD groups (*P* < 0.05). A total of 12 metabolic pathways were significantly affected by supplementation, including the signaling pathways of glucagon, insulin, and PI3K-AKT; primary and secondary bile acid biosynthesis; and P-type Ca 2+ transporters (*P* < 0.05). Hence, the **CbD** supplementation modulates bone metabolism by regulating the mediators of gut–brain axis, which may inform strategies to prevent leg diseases and improve meat quality in broilers.

## INTRODUCTION

Being one of the major sources of animal protein, demand for poultry has been increasing for decades. This has resulted in a drive to produce fast-growing, large broilers with good feed conversion rates, and high growth rates (Havenstein et al., 1994). The incubation temperature, transports time, feeding mode, body weight, and growth rate of broilers can affect their leg strength, walking ability, and then meat quality (Knowles et al., 2008; Güz et al., 2020). Under current practices, these factors combine to yield various negative health consequences, such as the development of contact dermatitis, arthritis, tenosynovitis, and claudication (Santos et al., 2022). Leg disorders are closely associated with poor growth and feed conversion, high mortality rate, carcass condemnation, and low meat quality (Oviedo-Rondón et al., 2009). In the USA, lameness and bone diseases in broilers cost manufacturers more than $150 million annually (Kierończyk et al., 2017). Therefore, leg disorders in broilers during poultry production deserve more research attention.

Several studies have recently reported that the intestinal microbiota is closely related to bone health (Yan et al., 2016; Li et al., 2020). Specifically, changes in the intestinal microbiota can disrupt the normal functioning of the gastrointestinal tract and affect the growth and development of bones. Ohlsson et al. (2017) found that intestinal microorganisms activate the expression of receptor activator for nuclear factor-κb ligand (**RANKL**) and tumor necrosis factor-α (**TNF-α**) in mice, which induce the osteoclast formation and bone resorption. Furthermore, Mohammed et al. (2021) has confirmed the positive effects of probiotics on broilers’ bone development. Schwarzer et al. (2016) found that *Lactobacillus plantarum* supplementation promotes juvenile growth, and prevents developmental retardation in male CONV-R germ-free mice during chronic malnutrition. Oral treatment with *Lactobacillus reuteri* also improves dynamic measurements of the distal femur epiphysis, lumbar trabecular parameters, osteoblast serum markers, and bone formation in male mice (Mccabe et al., 2013).

Several factors involved in the gut–brain axis—including intestinal microorganisms, short-chain fatty acids (**SCFAs**), and 5-hydroxytryptamine (**5-HT**)—play important roles in regulating bone development. Yan et al. revealed that GF mice chronically colonized with microbiota show improved bone formation owing to the enhanced secretion of insulin-like growth factor 1 (**IGF-1**) (Yan et al., 2016). The gut microbiome also increases butyrate levels, which allows the parathyroid hormone (PTH) to boost Treg levels, thereby preventing bone loss (Li et al., 2020). The monoamine hormone and neurotransmitter, 5-HT, modulates bone remodeling and promotes bone formation (Lavoie et al., 2017). SCFAs not only prevent bone loss but also regulate the activity of bone cells and affect bone healing (Wallimann et al., 2021). Notably, *C. butyricum* has been shown to produce SCFAs, regulate the gut microbiota, and play a positive role in the gut–brain axis (Sun et al., 2021). In addition, commercially available vitamin D (25-OH-D_3_) is an important feed additive that enhances bone development in broilers. However, it has some limitations in improving bone-related problems in birds during broiler production (Mitchell et al., 1997).

In a previous study, we showed that *C. butyricum* modulates the cecal microbiota, enhances butyrate production, and promotes bone development in broilers during the early stages of growth (Yu et al., 2022). Based on our findings, we hypothesized that *C. butyricum* regulates the gastrointestinal microbial structure of broilers and affects the secretion of hormones, brain–gut peptides, and neurotransmitters by changing the SCFA-mediated enteric-brain axis. Through this mechanism, *C. butyricum* may affect bone metabolism and prevent leg diseases in broilers. We also hypothesized that *C. butyricum*, when used in combination with 25-OH-D_3_, may have an additive effect in mitigating skeletal problems. To test these hypotheses, we evaluated how *C. butyricum* (alone or in combination with 25-OH-D_3_) affected the growth, intestinal flora, and bone metabolism of broilers.

## MATERIALS AND METHODS

### Animal Management and Sample Collection

In total, 800 one-d-old male Cobb birds (obtained from Zhejiang Guangda Breeding Poultry Co., Ltd, Hangzhou, China) were randomized into 5 treatment groups (8 replicates, with 20 birds per replicate). The groups were fed the following diets: basal diet (**Con**), basal diet + 75 mg/kg chlortetracycline (**Anti**), basal diet + *C. butyricum* (CGMCC 9386) at 10^9^ CFU/kg (**Cb**), basal diet + *C. butyricum* at 10^9^ CFU/kg and 25-OH-D_3_ at 25 μg/kg (**CbD**), and basal diet + 25-OH-D_3_ at 25 μg/kg (**CD**). In the building in which the birds were housed, the temperature was gradually reduced from 35 to 29°C at a rate of 2°C/wk. The feeding experiment was conducted for 42 d, feed and drinking water were provided ad libitum. Food intake and mortality were recorded daily. This study strictly observed the rules of the Ministry of Health of the People's Republic of China, and the protocol was approved by the Animal Care and Use Committee of Zhejiang A&F University (ZAFUAC 2023043, Hangzhou, China). The basic nutritional levels and compositions of the diets followed NRC (1994) recommendations and are listed in Table 1. The probiotic and 25-OH-D_3_ used in the present study were provided by Zhejiang Vegamax Biotechnology Co., Ltd (Anji, China).

**Table 1:** Composition and nutrient levels of the basal experimental diet (air-dry basis).

Items	Ages (days)	
	1–21	22–42
Ingredients (%)		
Corn	56.33	57.4
Soybean meal	24.5	19
Extruded soybean	5	4
Distillers dried grains with solubles	5	8
Corn gluten meal	2	3
Soybean oil	1.2	4.3
Limestone	1.3	1.3
Fermented soybean meal	1.67	0
Premix^1^^,^^2^	3	3
Total	100.00	100.00
Nutrient levels		
Metabolizable energy (kcal/kg)	2949	3148
Crude protein (%)	20.6	18.6
Crude fat (%)	4.9	8.0
Lysine (%)	1.17	0.99
Methionine+Cysteine (%)	1.45	1.23
Threonine+Tryptophan (%)	1.13	0.95
Calcium (%)	0.88	0.79
Total phosphorus (%)	0.64	0.56
Available phosphorus (%)	0.39	0.34

1 Minimal vitamin levels per kg of diets: vitamin A (retinyl acetate), 1,500 IU; vitamin D_3_ (cholecalciferol), 2,00 IU; vitamin E (*DL*-α-tocopheryl acetate), 10 IU; riboflavin, 3.5 mg; pantothenic acid, 10 mg; niacin, 30 mg; cobalamin, 10 μg; choline chloride, 1,000 mg; biotin, 0.15 mg; folic acid, 0.5 mg; thiamine 1.5 mg; pyridoxine 3.0 mg.

2 Minimal mineral levels per kg of diet: Fe, 80.00 mg; Cu, 8.00 mg; Mn, 60.00 mg; Zn, 40.00 mg; I, 0.18 mg; Se, 0.15 mg.

On d 42, one bird per replicate was selected for sample collection. Blood was sampled from the inferior wing vein of selected birds, following which the birds were peacefully euthanized. The blood samples were centrifuged at 3,000 × *g* (25℃, 15 min) for serum collection, which serum was stored under -80°C until further use. Aseptic cryopreservation tubes and scissors were used to collect the cecal contents of birds. All cecal samples were stored at -80°C until SCFA analysis and microbial sequencing. The left tibia was manually defleshed, detaching the articular cartilage from the fresh bone, and then stored at -20°C, which all surrounding tissues were removed prior to examination of bone characteristics.

### Growth Performance

Body weight was recorded on d 1, 21, and 42, and feed consumption was daily recorded, which determine the average daily feed intake (**ADFI**), average daily gain (**ADG**), and feed-to-body weight gain ratio (**F/G**).

### Tibial Indices

The adherent muscles and other soft tissues were removed from the tibia, and the length, width, and weight of the bone were measured. Bone radio graphic density was measured as described in a previous study (Deng et al., 2010). The anteroposterior view was used to acquire radiographic images of the tibia (2.5 mAs; 45 kV). Following the methods used by Zhou et al. (2009), the bone index was calculated as: bone index (g/kg) = tibial mass (g)/body mass at slaughter (kg). The length was determined from lateral intercondylar tubercle to inferior articular surface.

Bone-breaking strength was determined via a 3-point flexural bending test, following the methods outlined by Fleming et al. (Fleming et al., 1998). Each sample was kept at the same position, and held by cradle support. A 5kN load cell (20 mm/s speed) was imposed at the intermediate point, and the software Nexygen Plus (Lloyd Instruments, UK) was used for recording the bone-breaking strength (N) after suitable calibration.

### Measurement of Ca and P Concentrations in Tibial Ash

The tibial samples were boiled, and all tissues and cartilage caps were removed as described in a previous study (Zhang, 2007). The Ca content of the bone was determined via the potassium ferrate method, and the P content was determined spectrophotometrically.

### Measurement of Latency-to-Lie Time

Following the methods of Groves et al. (Groves and Muir, 2017), LTL was measured on d 37. Birds were placed in a 50 × 35 × 50 cm^3^ plastic basin containing water at a depth of approximately 3 cm and a temperature of approximately 32°C. The latency was recorded as either the time until the birds squatted down and their body touched the water or 5 min.

### Bone Development-Related Indicators

Commercially available ELISA kits (Cusabio, Wuhan, China) were used to detect the serum levels of bone formation-related markers (alkaline phosphatase [**AKP**], total procollagen type I *N*-terminal propeptide [**TPINP**], osteocalcin [**BGP**], and β-crosslaps) and bone metabolism-related hormones (GLP-1, Peptide YY [**PYY**], serotonin [**5-HT**], dopamine [**DA**], PTH, IGF-1, and procalcitonin [**PCT**]) following the manufacturer's instructions. The specific commercial ELISA kits (Cusabio, Wuhan, China) to measure the contents of brain–gut peptides (5-HT, DA, GLP-1, and PYY) in the intestine (cecum and ileum) and of monoamine neurotransmitters (5-HT and DA) in the hypothalamus.

### Measurement of Cecal SCFA Levels

Cecal SCFA levels were measured using gas chromatography (**GC**) as described in a previous study (Cao et al., 2022), with minor changes. In short, 0.5 g of the cecal digesta from each bird was mixed with 1 mL of pre-cooled double-distilled water and centrifuged at 10,000 × *g* for 5 min at 4°C. The supernatant was then mixed with 25% metaphosphoric acid (v/v, 5:1), iced for approximately 0.5 h, and analyzed using an Agilent Technologies 7890B GC System (column dimensions: 30 m × 0.25 mm × 0.25 μm; Agilent Technologies, Palo Alto, CA).

### Metagenomic Analysis of Cecal Contents

Metagenomic analysis of the cecal contents (3 birds per treatment group) was performed by Shanghai OE Biotech Co., Ltd. (Shanghai, China). Trimmomatic software (v. 2.0) was used to remove low-quality reads from the DNA data. Metagenomic sequences were compared in batches, and species abundance was calculated based on the results of comparative analysis. Predicted protein-coding genes were functionally annotated by comparing their sequences with those in the KEGG (https://www.kegg.jp/), eggNOG v5.0 (http://eggnog5.embl.de/#/app/home), CAZyme (http://www.cazy.org/), and other databases. Thus, we used the total metagenomic dataset to elucidate the functions of the microbiome. Finally, KEGG pathway analysis was used to screen and analyze pathways related to bone metabolism.

### Statistical Analysis

IBM SPSS Statistics software (v. 26.0; IBM Corp., Armonk, NY) was used to analyze the data, and GraphPad Prism 8.0 (GraphPad Prism Inc., San Diego, CA) was used for data visualization. Measurements for each treatment group were compared using an analysis of variance (**ANOVA**) and Duncan's multiple range test and were expressed as the mean ± the standard error of mean. Differences between groups were considered statistically significant at the 5% level (*P* < 0.05).

## RESULTS

### Growth Performance

On d 21 and 42, the Anti, Cb, CbD, and CD supplementation significantly increased the BW of birds compared with Con treatment (*P* < 0.05, Table 2).

**Table 2:** Effects of *Clostridium butyricum* and 25-hydroxyvitamin D_3_ on growth performance and tibia parameters of broilers.

Item		Con	Anti	Cb	CbD	CD	*P*-value
BW/g	d 1	19.6 ± 0.75	19.5 ± 0.68	19.7 ± 0.59	19.5 ± 0.81	19.6 ± 0.75	0.68
	d 21	704.8 ± 42.32^b^	765.2 ± 45.14^a^	771.5 ± 54.28^a^	784.5 ± 50.36^a^	768.1 ± 56.31^a^	0.02
	d 42	3461.5 ± 92.18^b^	3881.6 ± 96.46^a^	3985.4 ± 91.33^a^	4109.5 ± 88.42^a^	3933.1 ± 86.53^a^	0.01
ADG/g	d 1–21	32.7 ± 0.46^b^	35.7 ± 0.24^a^	35.9 ± 0.56^a^	36.6 ± 0.90^a^	36.4 ± 0.53^a^	<0.01
	d 22–42	49.2 ± 1.04^c^	56.4 ± 1.07^b^	60.2 ± 1.83^ab^	61.7 ± 1.98^a^	58.3 ± 1.53^ab^	<0.01
	d 1–42	81.9 ± 0.69^c^	92.1 ± 0.94^b^	96.1 ± 1.94^ab^	98.3 ± 2.38^a^	94.8 ± 1.54^ab^	<0.01
ADFI/g	d 1–21	53.2 ± 0.73^b^	54.9 ± 0.40^ab^	55.9 ± 0.90^ab^	59.6 ± 1.55^a^	56.3 ± 0.89^ab^	0.01
	d 22–42	103.7 ± 2.14^b^	111.2 ± 2.11^ab^	117.3 ± 3.55^a^	120.6 ± 3.69^a^	114.8 ± 3.23^a^	0.04
	d 1–42	156.9 ± 1.60^c^	166.0 ± 1.89^b^	173.2 ± 3.67^ab^	180.2 ± 4.22^a^	171.2 ± 3.07^ab^	<0.01
F/G	d 1–21	1.63 ± 0.04^a^	1.54 ± 0.02^b^	1.56 ± 0.01^b^	1.63 ± 0.02^a^	1.55 ± 0.01^b^	0.03
	d 22–42	2.11 ± 0.03^a^	1.97 ± 0.02^b^	1.95 ± 0.04^b^	1.97 ± 0.07^b^	1.97 ± 0.02^b^	0.03
	d 1–42	1.92 ± 0.01^a^	1.80 ± 0.00^b^	1.80 ± 0.01^b^	1.83 ± 0.01^b^	1.81 ± 0.01^b^	<0.01
Tibia length / cm		9.38 ± 0.14^b^	9.29 ± 0.09^b^	9.57 ± 0.11^ab^	9.50 ± 0.09^ab^	9.74 ± 0.06^a^	0.03
Tibia width/cm		0.82 ± 0.02	0.79 ± 0.02	0.84 ± 0.03	0.81 ± 0.01	0.86 ± 0.02	0.22
Tibia weight/g		6.36 ± 0.44^ab^	5.36 ± 0.18^b^	6.89 ± 0.54^a^	6.03 ± 0.72^ab^	6.47 ± 0.16^a^	0.04
Tibia index/g/kg		2.97 ± 0.17	2.71 ± 0.17	3.06 ± 0.27	2.71 ± 0.20	3.28 ± 0.37	0.43

Con birds fed basal diet without any antibiotics and additive, Anti birds fed basal diet added with antibiotic, Cb birds fed basal diet added with *Clostridium butyricum*, CbD birds fed basal diet added with *Clostridium butyricum* and 25-OH-D_3_, CD birds fed basal diet added with 25-OH-D_3_. The data are presented as mean ± SEM (N = 8). Values in the same row with different letters are statistically significant (*P* < 0.05).

From d 1 to 21, ADG was significantly higher in the Anti, Cb, CbD, and CD groups than in the Con group (*P* < 0.05, Table 2); no significant difference was observed in ADG across these treatment groups. Moreover, CbD supplementation significantly increased the ADFI relative to the Con group (*P* < 0.05). F/G values were significantly lower in the Anti, Cb, and CD groups than in the Con, and CbD group (*P* < 0.05).

From d 22 to 42, ADG was significantly higher in the Anti, Cb, CbD, and CD supplementation groups than in the Con group (*P* < 0.05); F/G values were significantly lower in the Anti, Cb, CbD, and CD groups than in the Con group (*P* < 0.05). Moreover, the Cb, CbD, and CD treatments showed a significant improvement in ADFI relative to the Con group (*P* < 0.05). And, the CbD treatment significantly increased the ADG compare with Anti treatment (*P* < 0.05).

From d 1 to 42, Anti, Cb, CbD, and CD treatment significantly increased the ADG of birds, and decreased the F/G values compared with Con treatment (*P* < 0.05). And, the Cb, CbD, and CD treatments showed a significant increment in ADFI relative to the Con group (*P* < 0.05). And, the CbD treatment significantly increased the ADG and ADFI compare with Anti treatment (*P* < 0.05).

### Tibial Parameters

Compared with birds in the Con and Anti groups, birds in the CD group had significantly longer tibias on average (*P* < 0.05; Table 2). Tibial length was significantly greater in the CD group than in the Con and Anti groups (*P* < 0.05). In addition, Cb and CD supplementation significantly increased tibial weight relative to the Anti group (*P* < 0.05). No significant inter-group differences were observed in the other tibial indices.

### LTL, Tibial Ca and P Contents, and BMD and Bone-Breaking Strength

**Figure 1:**
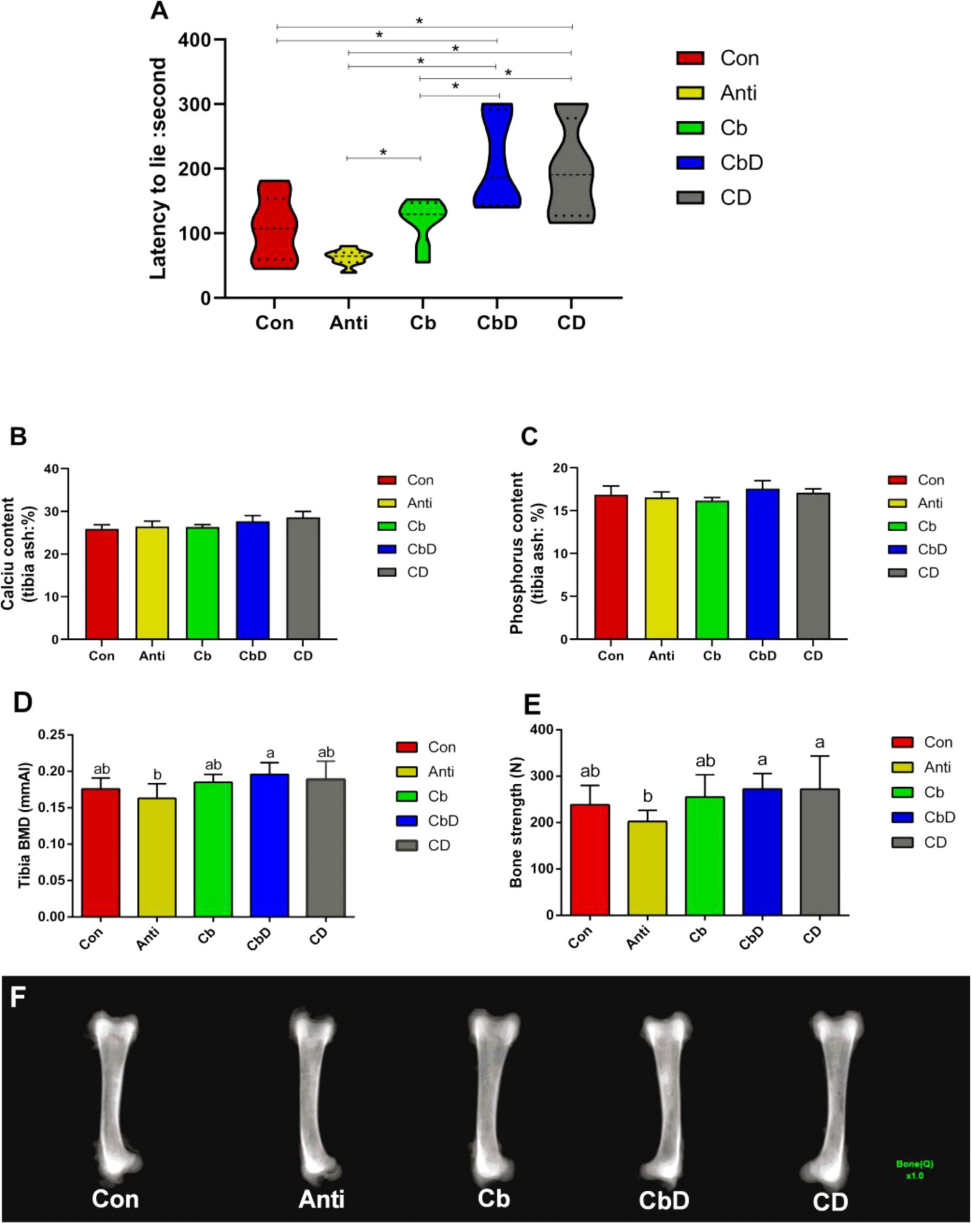
Effects of *C. butyricum* and 25-hydroxyvitamin D_3_ on the latency-to-lie time, tibial content of calcium and phosphorus, BMD and bone-breaking strengthen of broilers. (A) LTL. (B) calcium. (C) phosphorus. (D) BMD. (E) bone-breaking strengthen. (F) bone radiograph. Con birds fed basal diet without any antibiotics and additive, Anti birds fed basal diet added with antibiotic, Cb birds fed basal diet added with *Clostridium butyricum*, CbD birds fed basal diet added with *Clostridium butyricum* and 25-OH-D_3_, CD birds fed basal diet added with 25-OH-D_3_. Bars represent mean ± SEM (N = 8). * represents *P*<0.05.

On average, LTL times were significantly longer for birds in the CbD and CD groups than for those in the Con, Anti and Cb groups (*P* < 0.05; Figure 1A). Moreover, the average LTL times for birds in the CbD and CD groups were significantly longer than those for Cb-group birds (*P* < 0.05). The Cb group also had a significantly (*P* < 0.05) longer average LTL time than the Anti group. However, no significant inter-group differences were observed in the Ca and P contents of tibial ash (all *P* > 0.05; Figures 1B and 1C).

Compared with those in the Anti group, CbD-group birds had significantly higher tibial BMD of broilers (*P* < 0.05), and both CbD and CD-group birds had significantly (*P* < 0.05) higher bone-breaking strength (Figures 1D and 1E). The bone radiograph of all birds’ tibia was shown in Figure 1F.

### Bone Formation-Related Markers

**Figure 2:**
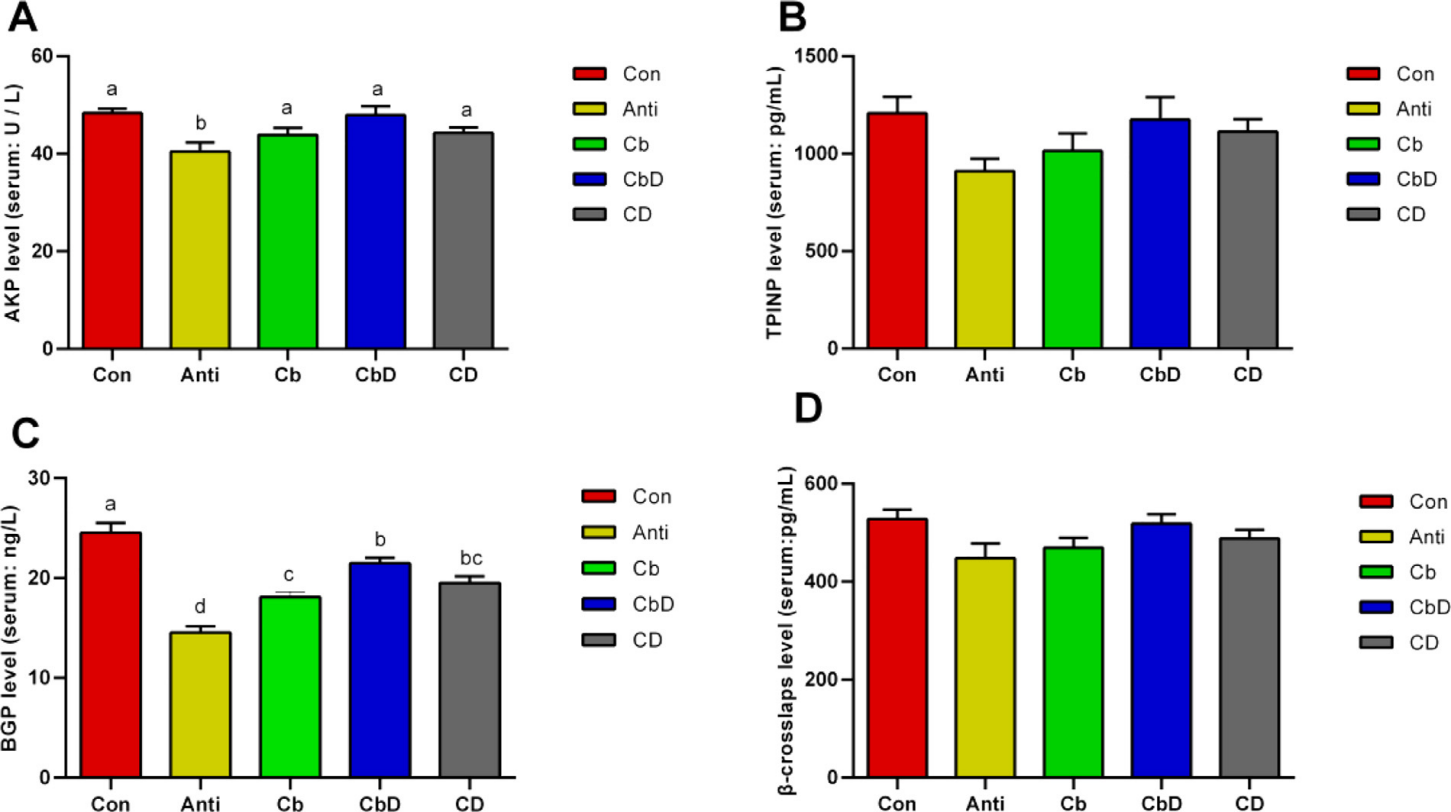
Effects of *Clostridium butyricum* and 25-hydroxyvitamin D_3_ on markers of bone formation in broilers’ serum. (A) AKP level. (B) TPINP level. (C) BGP level. (D) β-crosslaps level. Con birds fed basal diet without any antibiotics and additives, Anti birds fed basal diet added with antibiotic, Cb birds fed basal diet added with *Clostridium butyricum*, CbD birds fed basal diet added with *Clostridium butyricum* and 25-OH-D_3_, CD birds fed basal diet added with 25-OH-D_3_. Different lowercase letters above bars represent significantly different means *P* < 0.05. Bars represent mean ± SEM (N = 8).

The AKP concentrations were significantly higher among birds in the Cb, CbD, and CD groups than among those in the Anti group (*P* < 0.05; Figure 2A). No significant inter-group differences were observed in serum levels of β-crosslaps, and TPINP (*P* > 0.05; Figures 2B and 2D). Birds in the Anti, Cb, CbD, and CD groups had significant lower serum concentrations of BGP compared with those in the Con group (*P* < 0.05; Figure 2C).

### Bone Metabolism-Related Hormones

**Figure 3:**
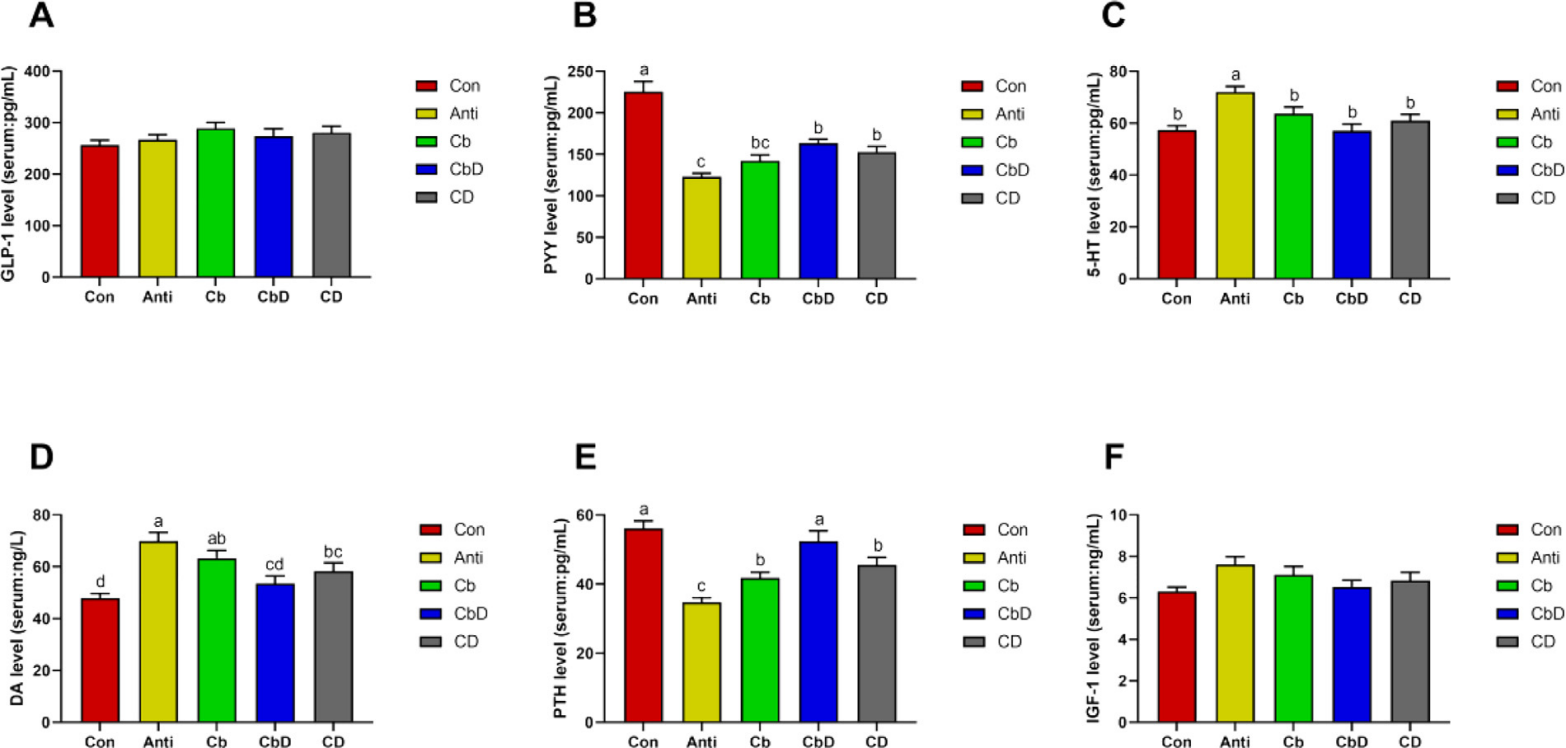
Effects of *Clostridium butyricum* and 25-hydroxyvitamin D_3_ on hormones related to bone metabolism in broilers serum. (A) GLP -1 concentration. (B) PYY concentration. (C) 5-HT concentration. (D) DA concentration. (E) PTH concentration. (F) IGF-1 concentration. Con birds fed basal diet without any antibiotics and additive, Anti birds fed basal diet added with antibiotic, Cb birds fed basal diet added with *Clostridium butyricum*, CbD birds fed basal diet added with *Clostridium butyricum* and 25-OH-D_3_, CD birds fed basal diet added with 25-OH-D_3_. Different lowercase letters above bars represent significantly different means *P*< 0.05. (N = 8).

No significant difference about the ileal GLP-1 was found among all birds (*P* > 0.05; Figure 3A). The Anti, Cb, and CD groups had significantly (*P* < 0.05) lower levels of serum PYY and PTH than the Con group (*P* < 0.05; Figures 3B and 3E). In addition, the serum PYY level was significantly (*P* < 0.05) lower in CbD than in the Con group. Serum 5-HT levels were significantly lower in the Cb, CbD, and CD groups than in the Anti group (*P* < 0.05; Figure 3C). Additionally, serum DA levels were significantly higher in the Anti, Cb, and CD groups than in the Con group (*P* < 0.05; Figure 3D). There were no significant inter-group differences (*P* > 0.05) in serum IGF-1 levels (*P* > 0.05; Figure 3F).

### Brain–Gut Peptides

**Figure 4:**
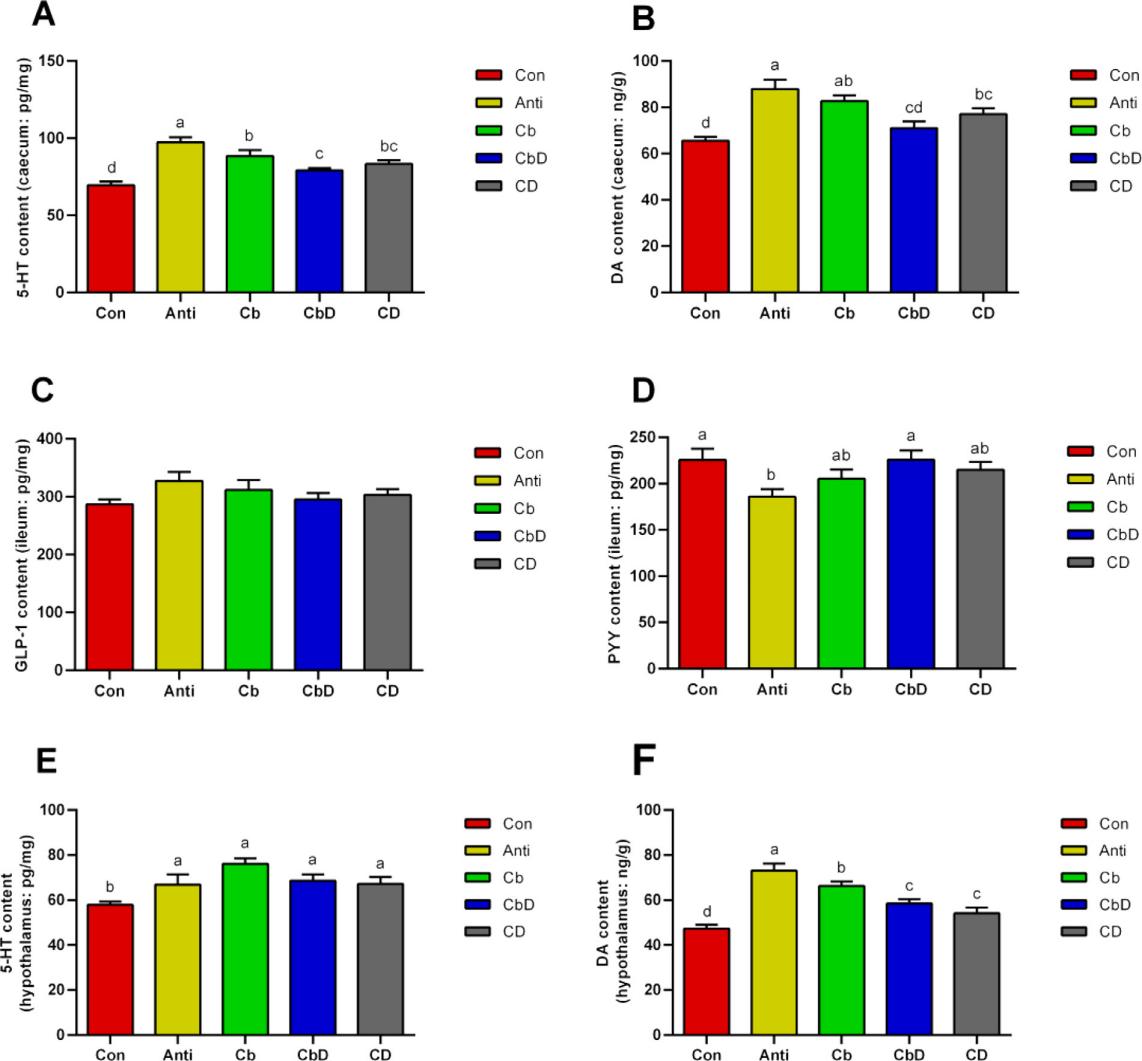
Effects of *Clostridium butyricum* and 25-hydroxyvitamin D_3_ on the hypothalamic and intestinal brain-gut peptides in broilers. (A) Caecal 5-HT content. (B) Caecal DA content. (C) Caecal GLP-1 content. (D) Ileal PYY content. (E) Hypothalamic 5-HT content. (F) Hypothalamic DA content. Con birds fed basal diet without any antibiotics and additive, Anti birds fed basal diet added with antibiotic, Cb birds fed basal diet added with *Clostridium butyricum*, CbD birds fed basal diet added with *Clostridium butyricum* and 25-OH-D_3_, CD birds fed basal diet added with 25-OH-D_3_. Different lowercase letters above bars represent significantly different means *P*< 0.05. (N = 8).

Caecal levels of 5-HT and DA were significantly higher in Anti, and CD group birds than in Con- and CbD-group birds (*P* < 0.05; Figures 4A and 4B). No significant i difference was found about the serum IGF-1 levels (*P* > 0.05; Figure 4C). Moreover, PYY levels were significantly higher in the CbD and Con group than in the Anti group (*P* < 0.05; Figure 4D). Hypothalamic 5-HT and DA levels were significantly higher in the Anti, Cb, CbD, and CD groups than in the Con group (*P* < 0.05; Figures 4E and 4F).

### SCFA Content of Cecal Digesta

**Figure 5:**
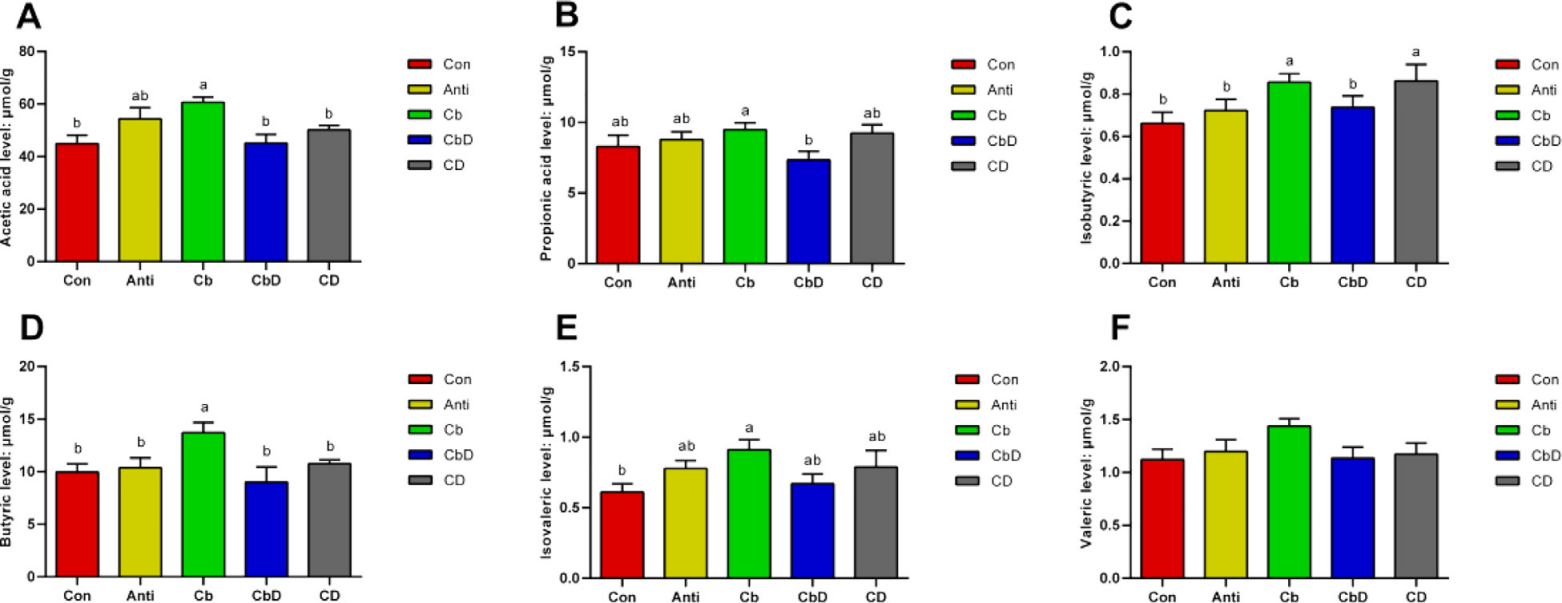
Effects of *Clostridium butyricum* and 25-hydroxyvitamin D_3_ on caecal SCFAs in broilers. (A) acetic acid level. (B) propionic acid level. (C) isobutyric level. (D) butyric level. (E) isovaleric level. (F) valeric level. Con birds fed basal diet without any antibiotics and additive, Anti birds fed basal diet added with antibiotic, Cb birds fed basal diet added with *Clostridium butyricum*, CbD birds fed basal diet added with *Clostridium butyricum* and 25-OH-D_3_, CD birds fed basal diet added with 25-OH-D_3_. Different lowercase letters above bars represent significantly different means *P* < 0.05. (N = 8).

Birds in the Cb group had significantly higher concentrations of acetic acid, isobutyric acid, butyric acid, and isovaleric acid in their cecal digesta than birds in the Con group (*P* < 0.05; Figures 5A, 5C–5E). Relative to the Con group, the Cb group had significantly lower levels of propionic acid (*P* < 0.05; Figure 5B). Levels of isobutyric acid and butyric acid were significantly higher in the Cb group than in the Anti group (*P* < 0.05; Figures 5C and 5D).

### Composition of the Gut Microbiota

**Figure 6:**
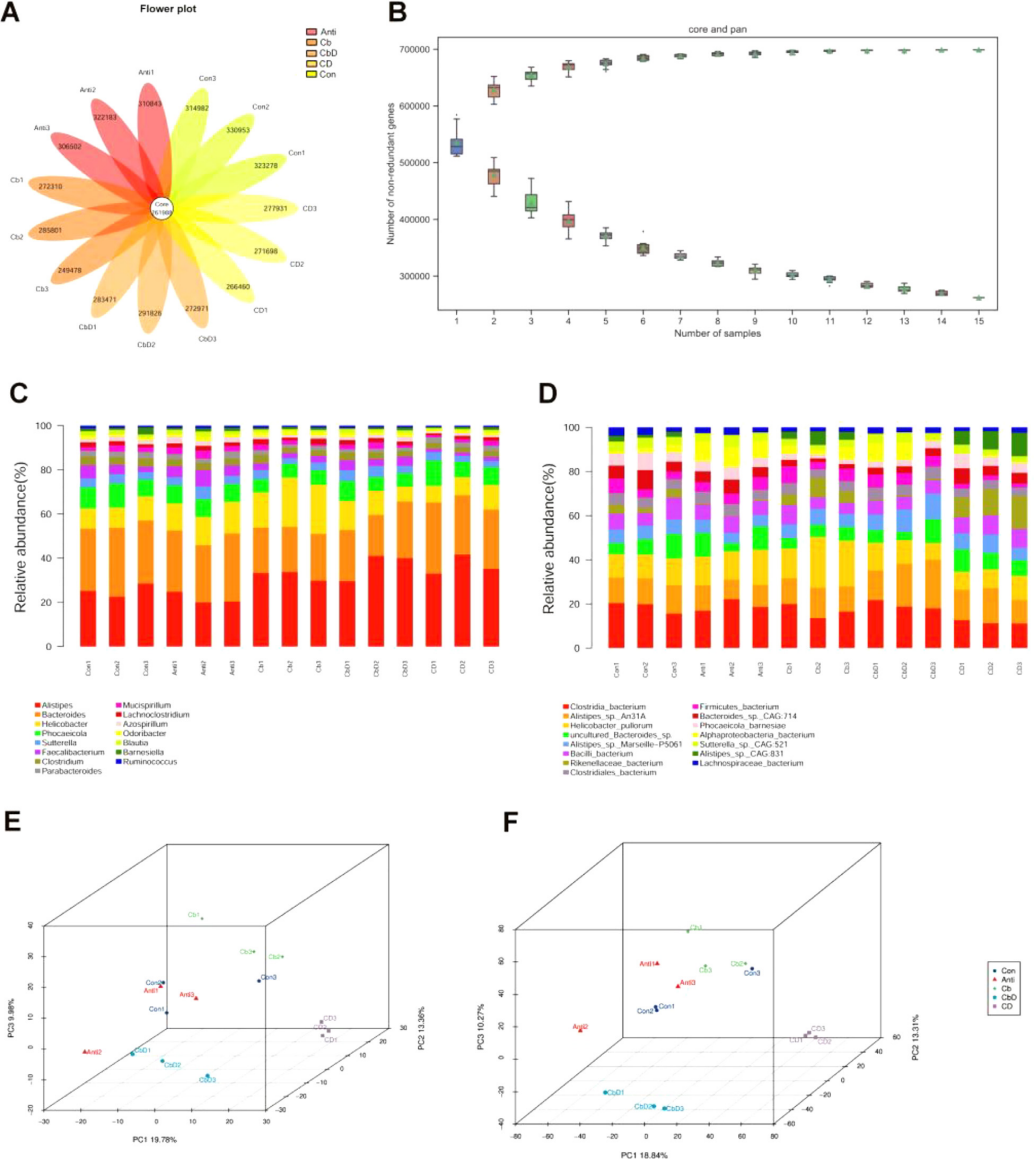
Effects of *Clostridium butyricum* and 25-hydroxyvitamin D_3_ on metagenome of broilers’ caecal microflora. (A) gene number venn graph. (B) core_Pan gene dilution curve. (C) species relative abundance histogram display based on genus level. (D) species relative abundance histogram display based on specie level. (E) PCA plot based on genus level. (F) PCoA plot based on specie level. Con birds fed basal diet without any antibiotics and additive, Anti birds fed basal diet added with antibiotic, Cb birds fed basal diet added with *Clostridium butyricum*, CbD birds fed basal diet added with *Clostridium butyricum* and 25-OH-D_3_, CD birds fed basal diet added with 25-OH-D_3_. (N = 3).

In total, 261,988 genes were common across all samples; however, the Con and Anti groups had more unique genes than the other three groups (Figure 6A). The dilution curve indicated that with an increase in the number of samples extracted (on the abscissa), the number of core/pan genes extracted tended to reach saturation (on the ordinate; Figure 6B). In terms of community composition, the top 12 genera in all the treatment groups included *Alistipes, Bacteroides, Helicobacter, Phocaeicola, Sutterella, Faecalibacterium*, and *Clostridium*. Bacteria in the class Clostridia, *Alistipes* sp*.* An31A, *Helicobacter pullorum*, uncultured *Bacteroides sp., Alistipes* sp*. Marseille-*P5061, *Bacillus* spp*.*, and bacteria in the family Rikenellaceae were the predominant species (Figure 6C). The abundance of *Alistipes* spp. at the genus level in Cb (17.2%), CbD (20.6%), and CD (20.6%) treatments’ birds were higher than those in the Con (13.8%) and Anti (11.2%). Moreover, the Cb group showed a significantly higher abundance of *Bacteroides* than the other groups. The Cb, CbD, and CD groups had a higher abundance of *Alistipes* sp*.* An31A than the Con and Anti groups. Furthermore, the abundance of *Helicobacter pullorum* was higher in Cb-treated birds than in the other groups (Figure 6D). PCA and PCoA analyses indicated that all samples from the Cb, CbD, and CD treatment groups were well separated from those collected from the Con and Anti treatment groups, suggesting that dietary supplementation in the Cb, CbD, and CD groups modulated the microfloral structure of broilers to a considerable degree (Figures 5E–5F).

**Figure 7:**
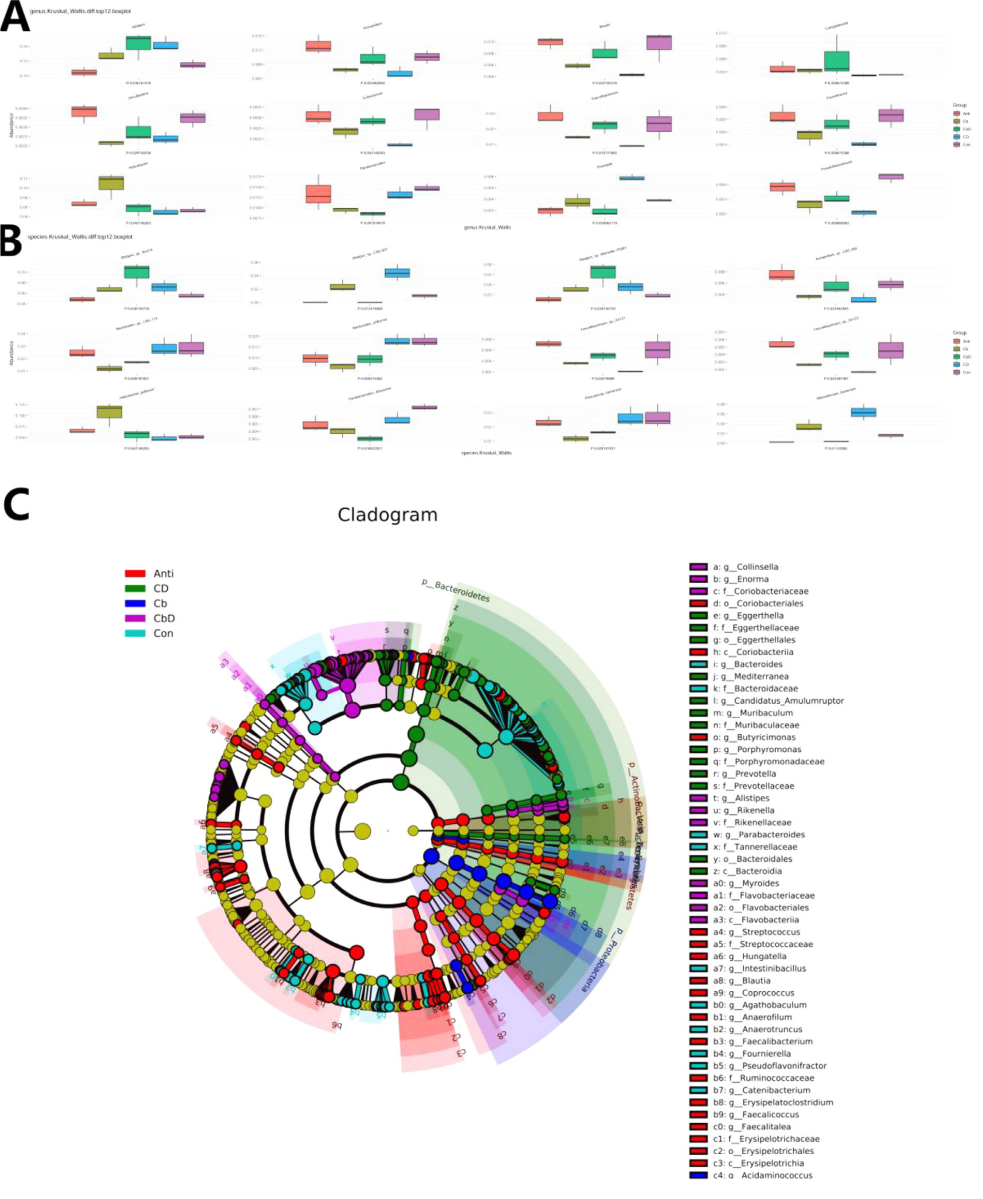
Effects of *Clostridium butyricum* and 25-hydroxyvitamin D_3_ on caecal microflora on genus and species levels in broilers. (A) top 12 distinguished genus based on Kruskal_Wallis analysis; (B) top 12 distinguished species based on Kruskal_Wallis analysis; (C) LEfSe analysis in genus level. Con birds fed basal diet without any antibiotics and additive, Anti birds fed basal diet added with antibiotic, Cb birds fed basal diet added with *Clostridium butyricum*, CbD birds fed basal diet added with *Clostridium butyricum* and 25-OH-D_3_, CD birds fed basal diet added with 25-OH-D_3_. (N = 3).

The top 12 genus- and species-level taxa were selected for multi-factor functional difference analysis (Figures 7A and 7B). At the genus level, the Cb, CbD, and CD groups showed a significantly (*P* < 0.05) higher abundance of *Alistipes* than the Con and Anti groups*.* Moreover, the abundances of *Azospirillum, Blautia, Desulfovibrio, Eubacterium, Faecalibacterium, Flavonifractor*, and *Pseudoflavonifractor* were significantly lower in Cb and CD-group birds than in those in the Con group. The abundance of *Parabacteroides* was significantly low in the CbD group (*P* < 0.05). The abundances of *Alistipes* sp. *An31A* and *Alistipes* sp. *Marseille-P5061* were significantly higher in the Cb and CbD groups than in the Con and Anti groups. The abundances of *Bacteroides* sp. *CAG:714, Bacteroides uniformis, Parabacteroides distasonis*, and *Phocaeicola barnesiae* were significantly low in the Cb and CbD groups. Moreover, the abundances of *Alistipes sp*. CAG:831 and *Rikenellaceae spp.* were significantly higher in the CD group than in the Con and Anti groups.

The results of LEfSe analysis showed that at the family level, Helicobacteraceae dominated the cecal microflora of birds in the Cb group; Eggerthellaceae, Porphyromonadaceae, Akkemansiaceae, and Prevotellaceae dominated in the CD group; Coriobacteriaceae, Flavobacteriales, and Campylobacteraceae dominated in the CbD group; and Streptococcaceae, Ruminococcaceae, Selenomonadales, Rhodospirillales, and Synergistaceae dominated in the Anti group (Figure 7C).

**Figure 8:**
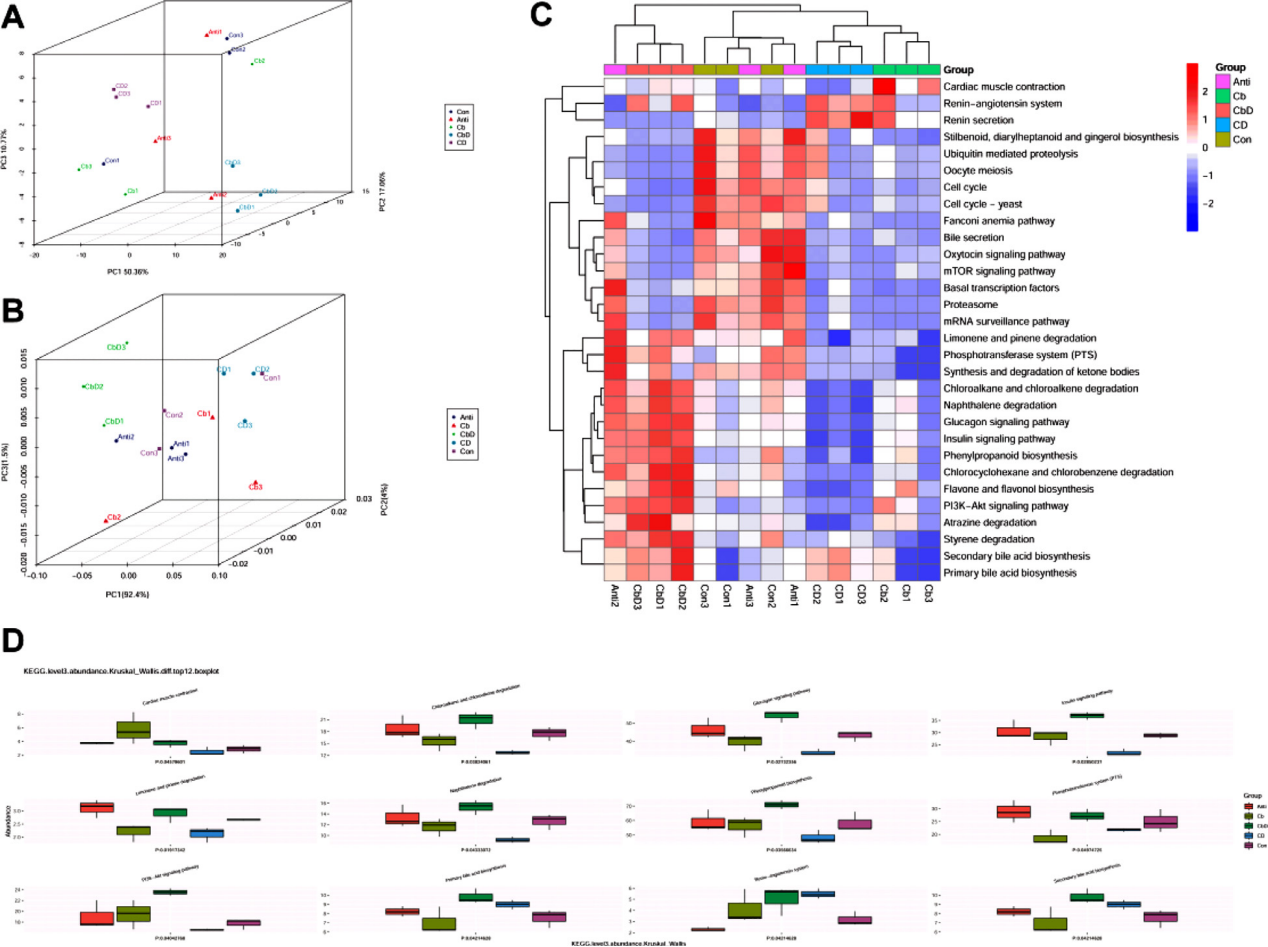
Effects of *Clostridium butyricum* and 25-hydroxyvitamin D_3_ on KEGG metabolic pathways of caecal microflora in broilers based on metagenomics. (A) Functional level PCA analysis. (B) Functional level PCoA analysis. (C) Heatmap of KEGG pathways. (D) Top 30 KEGG pathways abundance based on Kruskal–Wallis analysis. Con birds fed basal diet without any antibiotics and additive, Anti birds fed basal diet added with antibiotic, Cb birds fed basal diet added with *Clostridium butyricum*, CbD birds fed basal diet added with *Clostridium butyricum* and 25-OH-D_3_, CD birds fed basal diet added with 25-OH-D_3_. (N = 3).

Functional analyses with PCA and PCoA revealed that all samples collected from the experimental groups (Cb, CbD, and CD) were well separated from those belonging to the Con and Anti groups (Figures 8A and 8B). Visualization with a heat map confirmed that scores for metabolic pathways including the glucagon signaling pathway, naphthalene degradation, primary and secondary bile acid biosynthesis, insulin signaling pathway, phenylpropanoid biosynthesis, PI3K-AKT signaling pathway, and chloroalkane and chloroalkene degradation were higher in the CbD group than in the Con and Anti groups and lower in the CD and Cb groups (Figure 8C). Multi-factor comparative analysis of the top 12 KEGG pathways (level 3) revealed that the PI3K-AKT signaling pathway, primary and secondary bile acid biosynthesis, insulin signaling pathway, phenylpropanoid biosynthesis, glucagon signaling pathway, naphthalene degradation, and chloroalkane and chloroalkene degradation were significantly promoted after dietary supplementation in the CbD group (Figure 8D).

## DISCUSSION

Growth performance is the most basic and important indicator of broiler performance, and the positive effect of probiotics (when used as feed additives) on this measure broiler growth performance (Abdel-Hafeez et al., 2017). Studies have showed the growth promoting and immune enhancing effects of *C. butyricum* in broilers and piglets (Liao et al. 2015; Takahashi et al., 2018; Cao et al., 2019)*.* Our previous study also revealed that supplementation with *C. butyricum* in combination with 25-OH-D_3_ positively influenced the growth performance and bone metabolism of early broilers (Yu et al., 2022). The results of present study indicated that the Anti, Cb, CbD, and CD treatments significantly increased the BW and ADG compared with those in the Con treatmeng . In addition, the Con group had the highest F/G values among all groups. It is remarkable that the dietary combination of *C. butyricum* and 25-OH-D_3_ increased the ADG and ADFI than Anti supplementation. These findings are in accordance with those of other studies showing the positive effects of probiotics on broilers (Mohammed et al., 2021; Santos et al., 2022).

Bone health affects the welfare of broilers, and maintaining good bone health in birds strongly aligns with the economic interests of breeders (Shim et al., 2012). Supplementation with 1,25(OH)_2_D_3_ has been shown to improve bone ash content in broilers and improve cartilage dysplasia during different growth periods (Elliot et al., 1995). Similarly, supplementation with *B. subtilis* has been shown to significantly prolong LTL time, improve tibial length and weight, and improve the bone ash (Mohammed et al., 2021). Tibial bone mineral density (BMD) and ash content are susceptible to dietary Ca and P concentrations in broilers (Liu et al., 2017). In the present study, we found that CbD supplementation increased tibial length and BMD, LTL, and bone strength in broilers. Moreover, supplementation with 25-OH-D_3_ alone (CD) or Cb improved the tibial weight of broilers. Rizzoli and Biver (2020) reported that probiotics improve the growth performance and strength of tibial bones by increasing the digestibility of the diet and the resorption of Ca and P.

Serum levels of bone formation-related markers and hormones reflect the status of bone metabolism. Decreased levels of the propeptide of type I procollagen and osteocalcin have negative effects on bone structure and mineralization in broilers (Szulc et al., 2017). Moreover, gastrointestinal hormones are typically related to bone resorption-related markers and affect bone homeostasis (Jensen et al., 2021). Increased AKP activity is primarily related to increased osteoblastic activity in broilers, and can be treated as a positive marker to evaluate bone health (Asensio et al., 2020). The AKP activity of Cb, CbD and CD birds was higher than that of Anti birds, although wasn't different from that of Con birds in present study. The BGP regulates bone resorption, and is positively in close contact with mineralization process, and bone turnover (Niimi et al., 2014). Other hormones such as IGF-1, PCT, and PTH have also been shown to affect bone development (Dixit et al., 2021). Among them, the PTH facilitates bone turnover and reduces the loss of bone (Silva et al., 2011). In the present study, the Cb, CbD, and CD treatment groups had significantly lower levels of BGP than that of Con group, while higher than that of Anti group. In addition, the Anti, CB, and CD groups had significantly lower serum levels of PYY and PTH than the Con group; this indicated low levels of Ca in the blood, likely due to bone mineralization. Overall, the above results suggest that supplementation with *C. butyricum* and 25-OH-D_3_ changed the levels of bone formation-related markers and bone metabolism-related hormones, while more trials should be conducted to investigate the previous mechanisms.

Central 5-HT promotes bone development, prevents bone resorption, and increases bone mass in mouse (Yadav et al., 2009). Our data showed that the treatments with Cb, CbD, and CD increased the hypothalamic and cecal levels of 5-HT. In addition, the DA promotes bone remodeling and formation via the hypothalamic-pituitary-gonadal axis, and inhibits osteoclast differentiation via the cAMP/PKA/CREB pathway (Wang et al., 2021). Moreover, the Cb and CD treatments increased serum and intestinal levels of DA. Combined with the above studies, our data also suggest that the CbD treatment increased the production of 5-HT and DA, thereby improving bone mass. And, its regulatory effects on DA were more significant than those on 5-HT.

SCFAs produced by intestinal microorganisms enhance the expression of tryptophan hydroxylase 1, which promotes the synthesis of 5-HT by intestinal chromaffin cells (Reigstad et al., 2015). SCFAs also promote the secretion of IGF-1, and regulate bone mass in mice (Yan et al., 2016). In the present study, dietary supplementation in the Cb group significantly increased the levels of the major SCFAs, which is inconsistent with the results of previous studies. Hence, we speculated that Cb regulates bone mass via the modulation of neural DA by SCFAs. The production of SCFAs is closely related to the changes in bacterial flora. *Alistipes* is the main producer of bacterial SCFAs, and *Bacteroides* produces propionic acid (De Vadder et al., 2016). Our metagenomic analysis indicated that *Alistipes, Bacteroides, Faecalibacterium*, and *Clostridium* were the dominant genera in all treatment groups, and that treatment with Cb significantly increased the abundances of these bacteria.

Previous studies on glucagon, insulin, and bone metabolism have indicated that the metabolism of bile acid by intestinal microbes regulates host immunity (Zhao et al., 2017), which this process is closely tied to classical bone metabolism via related signaling pathways (Cui et al., 2020). DA interacts with the adrenergic system to regulate glucagon and insulin secretion via adrenergic and dopaminergic receptors (Aslanoglou et al., 2021). Therefore, supplemental *C. butyricum* may affect bone metabolism by regulating the structure of the intestinal microflora, and this may be accompanied by the upregulation of various bone metabolism-related pathways. KEGG pathway analysis revealed that the abundance of P-type Ca^2+^ transporters differed from that typically associated with the signaling pathways of glucagon, insulin, PI3K-AKT, and primary and secondary bile acid biosynthesis. This explains the potential mechanism through which supplementation with *C. butyricum* upregulated the expression of bone formation-related markers, bone metabolism-related hormones, and SCFAs and confirms our initial hypothesis. In addition, Fox et al. (2008) suggested that P-type Ca^2+^ channels regulate the release of neurotransmitters, and these channels are selectively inhibited by 5-HT (Ciranna et al., 1996). Therefore, supplemental *C. butyricum* may regulate bone metabolism via the neurotransmitter 5-HT, although the specific mechanism of this effect needs to be further explored at the cellular and molecular level.

## CONCLUSIONS

Our results suggest that supplementation with *C. butyricum* (at 10^9^ CFU/kg) and 25-OH-D_3_ (at 25 μg/kg) can improve growth performance of broilers by modulating the bone health and metabolism. Specifically, the combination of *C. butyricum* and 25-OH-D_3_ supplementation significantly increased the abundance of *Alistipes* and *Bacteroidesis,* the main strains producing SCFAs, and also modulated other mediators of the gut–brain axis (hormones and neurotransmitters). Our findings are of great importance for the broiler production industry, and may contribute to the development of novel management techniques to prevent leg diseases, thus reducing economic losses.

## DISCLOSURES

The authors declare no conflicts of interest.
